# Tracing the Disparity Between Healthcare Policy–Based Infrastructure and Health Belief–Lead Practices: a Narrative Review on Indigenous Populations of India

**DOI:** 10.1007/s40615-023-01810-3

**Published:** 2023-10-03

**Authors:** Biju Soman, Ashwathi Raj Lathika, B. Unnikrishnan, Ranjitha S Shetty

**Affiliations:** 1 Department of Community Medicine, Kasturba Medical College, Manipal, Manipal Academy of Higher Education, Manipal, Karnataka 576104 India; 2 Prasanna School of Public Health, Manipal Academy of Higher Education, Manipal, Karnataka 576104 India; 3 Department of Community Medicine, Kasturba Medical College, Mangalore, Manipal Academy of Higher Education, Manipal, Karnataka 575001 India; 4 Centre for Indigenous Population, Kasturba Medical College, Manipal, Manipal Academy of Higher Education, Manipal, Karnataka 576104 India

**Keywords:** Indigenous people, Scheduled Tribes, Health policy, Culture, Practices, Utilization, India

## Abstract

Most Indian tribes have limited access to healthcare facilities and rely heavily on traditional healing practices. This narrative review aimed to identify the disparities in the implementation of healthcare services and in accessing and availing these services by the indigenous population in India. We also have tried to throw light on the plausibility in strengthening the efficiency and efficacy of the public health system, by utilizing the available resources to its maximum potential, so that there will be a measurable outcome in the health status of these populations in India, coherently with the relevant sustainable development goals (SDG). The evidence from published literatures supports the fact that the disparity exists in the health status of indigenous populations in India as compared to the general populations. It emphasizes the need to address the key determinants such as the lack of knowledge, traditional healing practices and poor utilization of healthcare services provided to them. Various factors such as accessibility to healthcare resources, traditional healing practices, lack of awareness regarding healthcare services and schemes provided by the government, insufficient data regarding their issues and challenges and cultural and language barriers worsen the health status of indigenous people. However, our review reiterates that a well-structured and sustainable policy with reframed infrastructure and administration of healthcare system might bring a positive change in the health status of indigenous population in India.

## Introduction

India is the world’s most populous country, according to UN estimates [1]. India’s current population is around 1.4 billion people, with 104 million (8.6%) tribal people, made up of 52.5 million men and 52 million women [2]. The population of India’s Scheduled Tribes (STs) has been growing since 1961, with a growth rate of 21.3% in census 2011 compared to 2001 making it the world’s largest tribal population [3].

The tribal population occupies almost 15% of India’s total land area, with 91.7% living in rural areas and 8.3% in urban areas. Scheduled Tribes (ST) of India are defined as “Tribes or tribal communities or parts of or groups within tribal communities which the president of India may specify by public notification” [4]. The 705 tribal groups identified in the country are categorized as (a) tribal people living in Schedule-V areas (preponderance of tribal population, which should not be less than 50%, compactness and reasonable size of the area; underdeveloped nature of the area; and marked disparity in the economic standard of the people, as compared to the neighbouring areas) and tribal dominant blocks and districts, (b) tribal population in North-East India, (c) Particularly Vulnerable Tribal Groups (PVTG) and (d) Tribal people living outside scheduled areas [5]. The Government of India (GoI) acknowledged most vulnerable tribal groups, as a distinct category called Primitive Tribal Groups (PTGs), and declared 52 such groups in 1975, and another 23 groups were added to the category, making it a total of 75 PVTGs out of 705 STs in 1993. The majority of STs dwell in the states of Andhra Pradesh, Assam, Jharkhand, Gujarat, Chhattisgarh, Maharashtra, Orissa, Rajasthan and West Bengal [6].

These tribal communities differ in their beliefs, attitudes and practices, making them a heterogeneous group that cannot be lumped together under a single umbrella other than the shared terminology.

The alarming factors that should be an eye-opener for the policymakers and other stakeholders are the disparity in the healthcare services provided to the ST population in India. They have poor health and poor access to the healthcare facilities provided to them by the public sector [7]. A well-established plan, policy and execution of the healthcare delivery programs that are tailored to the requirements of the tribal population, would certainly bring their health status up to the level of general population.

## Aim of the Review

This narrative review aimed to identify the disparities in the implementation of healthcare services and in accessing and availing these services by the indigenous population in India. We also have tried to throw light on the plausibility in strengthening the efficiency and efficacy of the public health system, by utilizing the available resources to its maximum potential, so that there will be a measurable outcome in the health status of these populations in India, coherently with the relevant sustainable development goals (SDG).

## Methods

An extensive literature search was done to describe the health status of tribes in India, and the mismatch between the governance and implementation of healthcare services to the heterogenic tribes. We have used the Scale for the Assessment of Narrative Review Articles-SANRA, using categories 0–2 on a scale consisting of six items, to ensure the quality of this review [8]. All published articles on the data sources PubMed, Embase, Scopus Web of Science and Cochrane were used. The articles published in the past 32 years were considered (January 1990 to December 2022). The search terms used were “tribes, primitive tribes, tribal population, indigenous population, *adivasis*, scheduled tribes, tribal health, health policy, tribal health in India, tribal culture, barriers, healthcare”, considering the varying label of indigenous populations globally. We used the search terms individually and Boolean search strategies, including MeSH terms for this review in English language. Peer-reviewed articles and reports were extracted from the literature search, applying related words and equivalent subjects *Adivasi*, *indigenous population* and primitive tribes to extract studies from different regions and continents). A detailed presentation of search is depicted in Table 1.

**Table 1:** Literature search tracking sheet

Date of search	Source type	No. of hits	Search terms
19/01/2022	Academic journals	220	Tribal health in India and *adivasis*
19/01/2022	Reports	15	Health policy
02/02/2022	E books	40	Health policy
02/02/2022	Magazines	8	Tribal health
28/10/2022	Books	2	Tribals and health practice
08/06/2022	News	1	Health practices of tribes

This review is comprised of articles which were published in English language, between the years 1990 and 2022. Only peer-reviewed articles which discussed on tribal population, focused on healthcare services among tribes in India as well as other countries, were included. Articles which dealt with tribal health, health policies and tribal culture were considered for pooling the information. After excluding the literatures which were not directly focusing on tribal health, those which failed to provide meaningful evidence synthesis and inconsistent with the objective of this review, we considered only 33 literatures, including government reports (Table 2).

**Table 2:** Inclusion and exclusion criteria for the review of literature

Inclusion criteria	Exclusion criteria
Evidences referencing on health status of tribal population	Literature not directly focusing on tribal health and issues
Literatures focusing on healthcare services among tribes in India	Literature which has methodological inconsistency (eliminated by reading abstracts) in synthesis of evidence, such as unclear explanation on methodology which could not create adequate evidences, comments and opinion type articles.
Literature which were peer reviewed	Literature which was published in other than English
Articles from any continents, referencing to tribal health, health policies and their culture	
Articles published between January1990 and December 2022	
Government reports published from India between 1990 and 2022, on tribal health	

## Results

**Fig. 1:**
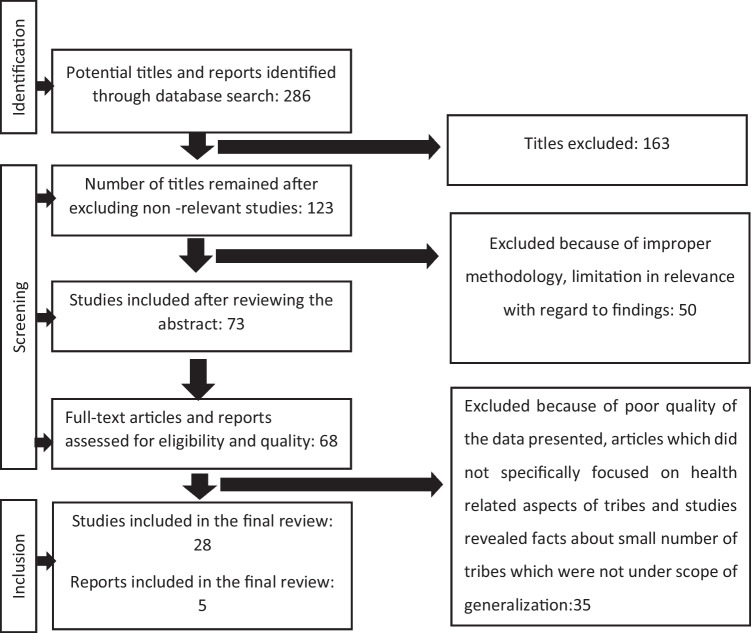
Flow diagram depicting literature selection

After searching for open access, peer-reviewed full-text articles and all resources, we got 286 literatures. After excluding the literatures which were not consistent with the objective of this review (shown in Table 2), 33 literatures were included for review (Fig. 1).

**Fig. 2:**
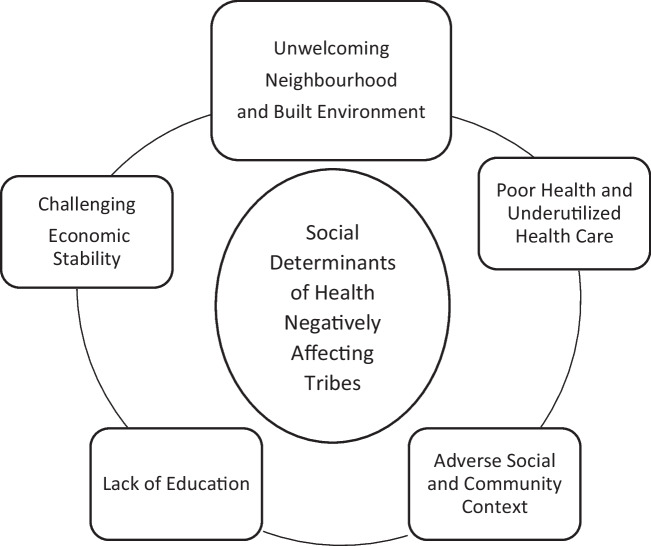
Social Determinants of Health (SDOH) model to analyze the adverse elements that contribute to under privileged state among tribes

Social Determinants of Health (SDOH) model was well integrated with the findings of this review. Boosting those five key elements in SDOH, which are neighbourhood and built environment, health and healthcare, social and community context, education and economic stability, can improve the health status of tribal population and lessen the disparity between the policy, administrative strategies and utilization of healthcare services by tribes (Fig. 2).

**Fig. 3:**
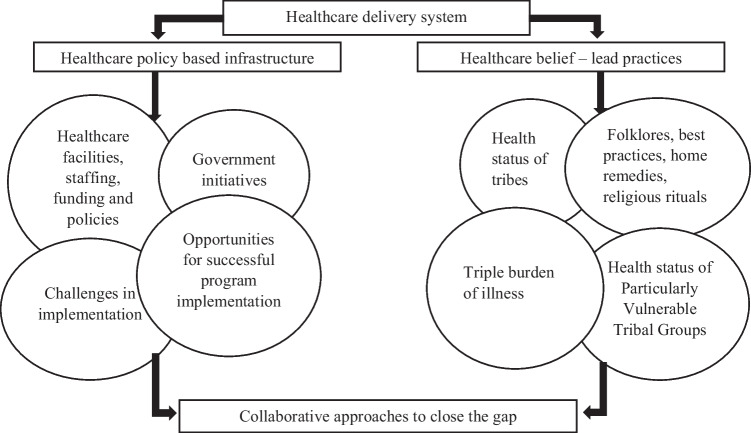
Framework of the key elements responsible for the disparity between healthcare policy–based infrastructure and health belief–lead practices

A framework was designed for the narrative review which categorizes the key elements in to “healthcare policy-based infrastructure” and “health belief–lead practices” (Fig. 3).

### Health Status of Indigenous Population

Indigenous population of India has poor health status and limited access to public healthcare services, even though nearly 50% of the total tribal population depend on public healthcare services in India. As per the available data, 40.6% of ST are below the poverty line while this proportion for general population is only 20.5% [6]. The vulnerable tribal groups who live in various isolated locations across India confront several health concerns and issues, many of which are linked to their culture and habits in addition to poverty [9].

The key health indicators are remarkably poor among tribes in India as compared to the non-tribal population. The National Family Health Survey 4 (NFHS-4/2015–2016) reported that the mortality of tribal children under 5 years of age was 57.2 per 1000 live births, whereas it was 38.5 per 1000 live births among others. Likewise, the Infant Mortality Rate (IMR) was 44.4 per 1000 live births as compared to 32.1 in the remaining population [10]. Health reports usually lay emphasis on the magnitude of these health issues as the child of a tribal family in India is at 19% higher risk of dying in the neonatal period and 45% in the post-neonatal period, as compared to their counterparts among the general population at any point of time [11]. However, enforcement of strategic interventions by the healthcare system has been always flaccid in tribal areas leading to no further improvement in their health status.

### Triple Burden of Illness

The triple burden of illness (communicable diseases, non-communicable diseases and emerging globalization-related health conditions) among tribal populations in India further makes it a difficult challenge for the government to tackle their health problems. This could be due to the neoliberal model of liberalized trade and investment which has created minimal government regulation.

Cases of tuberculosis (TB) have been found to be an alarming health issue in India’s diverging ST community, (an estimated PTB of 261 per 100,000 population in a year) afflicting many states [12, 13] and accounting for a significant portion of infectious diseases and healthcare cost burden among them. Faults in the public healthcare delivery system, particularly at the grassroots level among tribal communities, have been identified by several researchers and practitioners as a major factor to the rising TB caseload [14].

Rapid urbanization and changing lifestyles and surroundings have increased the prevalence of non-communicable diseases such as cancer, diabetes and hypertension, in addition to high rates of malnutrition and communicable diseases in the indigenous communities. Likewise, in indigenous communities, mental health issues such as stress, melancholy, anxiety, suicide ideation and addiction have also been documented [3]. A systematic review published in 2020 revealed that majority of tribal population in India are underprivileged in the mental healthcare services provided by the public healthcare system. Substance use and suicidal ideations are quite rampant among them and relying on traditional healing practices for these conditions is not a rare phenomenon in these communities. Poverty, peer pressure, cultural beliefs, stigma, stress and remote dwelling practices are the most significant barriers that limit their access to mainstream mental healthcare services [15].

Animal attacks, snake bites, violence, issues related to geographical terrain and distance, poor socio-economic index due to low levels of education, low income, poor housing conditions, lack of safe water supply and sanitation facilities are among the other issues that are reported to be prevalent among tribes which put their health status at major risk [16]. Even though the National Health Mission (NHM) aimed to establish functional health facilities in the public domain by revitalizing existing infrastructure in India, it was unable to make a significant improvement in the healthcare utilization of tribal populations due to disparities in resource allocation and utilisation, as well as tribe-specific health beliefs and behaviour [9].

A considerable proportion of indigenous people in India make a living by collecting forest goods, hunting wild animals, shifting farming, maintaining domesticated herds and doing handicrafts. Only 10.7% of the tribal people have access to safe drinking water, and 74.7% practice open-air defecation, which is clearly harmful to their community’s health. Only 6.7% of STs are reported to have finished 12 years of schooling, while more than one-third (41%) are illiterate. The average life expectancy at birth is 63.9 years (67 years for the general population). The main causes of a high Maternal Mortality Rate (MMR) are early marriage, early childbirth, undernutrition and a high incidence of anaemia, according to reports. The reports have documented that only 15% of pregnant women from tribal communities receive full antenatal care (ANC) and about a quarter of them (27%) still practice home delivery. Only 37% of tribal women receive post-natal care (PNC), within 48 h of childbirth, and this could be one of the reasons for higher IMR (74 per 1000 live births) in the tribal pockets of the country while it is 62 per 1000 live births for the rest of population. Mainly, cost of healthcare services and distance to be travelled are the hindering factors to utilize healthcare services in certain tribal areas especially where they depend mostly on private practitioners and small clinics. In addition to this, maternal services being provided by the government are not tailored to the tribe-specific health beliefs and cultural practices such as having trust in their own community persons in conducting deliveries and following the practices of home delivery [6].

### Health Infrastructure in Tribal Areas

Although prevailing community ethos, cultural beliefs and practices within tribal communities have formed a distinct barrier for the healthcare delivery to the tribal population in India in the past decades, changing attitude and practice of younger generations, influenced by the initiatives of non-governmental organizations (NGOs) and other governmental involvements, have enhanced the acceptance of modern medicine significantly in the recent years. However, language barriers, insufficient knowledge about many health schemes and services, and discrimination by the healthcare providers are still the major hindering factors for effective utilization of services by them [17].

According to the data released by Ministry of Health and Family Welfare (MoHFW) on the rural health infrastructure in tribal areas as on March 2022, there are 25,383 sub-centres, 3833 Primary Health Centres (PHC) and 960 Community Health Centres (CHC) and report a shortfall of 9357 sub-centres, 1559 PHCs and 372 CHCs in these areas. Madhya Pradesh followed by Rajasthan had the highest shortage of these facilities in tribal regions [18]. The biggest impediments in achieving the health indicators among STs in those states are a continual lack of manpower and poor infrastructure within the healthcare delivery system [19].

### Health Beliefs and Practices

​​​​​​The majority of tribal groups have their own set of behaviours that are based on their culture, socio-economic considerations, generational customs and current trends. Most of the tribal groups have their own subgroups, each of which follows its own culture and rituals. For instance, Bhil, Meena and Kathodi tribes of the state of Rajasthan in India use animals in their magico-religious and socio-culture practices [20], whereas the Irula, Muduga and Kurumba tribes of the state of Kerala in South India link their healing and health practices to the nature, their forefathers and food [21]. Health beliefs, specifically sentiments of self-efficacy, are linked to one’s perception of one’s capacity to do a specific behaviour [22]. Inequalities associated with the distribution of healthcare services to the tribal communities in India has resulted in poor access to those services by tribal people. Because of the tribal model of the aetiology of illnesses, superstitions linked with health and disease, a large number of researchers have focused on the health beliefs of indigenous people from rural and remote locations, as opposed to urban settings. Barriers are classified as those related to their attitude, values, beliefs, structural or geographical distribution, socio-economic status, language and communication in a cross-cultural country like India. Although the availability of scientific data on these barriers is limited in India, a few Australian literatures have evidence of belief in supernatural power and its outcome on ill health among tribal populations [23]. Likewise, the tribal population in Bangladesh, who share homogenous cultural practices of eastern India, seek treatment for malaria symptoms from traditional healers initially, which could be reformed by providing culturally specific healthcare services [24].

The healthcare-seeking behaviour of tribal people is driven by their cultural beliefs and practices. However, the very few divergent tribal communities from their ancestor practices and having more affinity with the other non-tribal populations have undergone cultural dilution considerably, in which the utilization of public and private healthcare services may be more than among their counterparts. Cultural practices of certain tribal groups such as *Gond*, *Rajgond*, *Madia* and *Pardhan* in Maharashtra state in India were seem to be the barriers in the successful implementation of insecticide program as their culture prevents spraying of insecticides in the households altars where deities are placed for religious rituals [25]. Furthermore, educational materials offered by NGOs and government organizations are ineffective for tribal groups since they are unable to comprehend them due to the high prevalence of illiteracy and stakeholders’ limited knowledge of tribal languages [26]. The traditional health-seeking behaviour of indigenous people shows a very strong affinity with the belief in supernatural interposition with the ill health continuum. It is crucial for healthcare professionals to appreciate the belief system of this population and its impact on the utilization of healthcare services and its outcomes. Researchers reiterate that the health professionals should consider the principles of mutual respect and understand the gaps and barriers in the healthcare system that is catering to these populations. Interventions in the tribal communities such as providing culturally sensitive healthcare services, training healthcare workers to communicate in respective tribe’s language, understanding the role of cultural interpreters and indigenous health advocates in providing these services and providing treatments inclusive of shared decision-making need to be explored further. In addition to these approaches, respecting their traditional medicines, acknowledging and incorporating their best practices and working with tribal healers to integrate traditional and modern medicine, and coordinating the programs with tribal communities and individuals for overcoming the cultural and individual barriers. Thus, we can provide cross-cultural and comprehensive care to tribal populations based on the sustainable development goals (SDG) one to eleven, aiming on no poverty, zero hunger, good health and well-being, quality education, gender equity, clean water and sanitation, affordable and clean energy, decent work and economic growth, industry innovation and infrastructure, reduced inequalities and sustainable cities and communities [27–30].

### Health Status of Particularly Vulnerable Tribal Groups

In India, 75 tribal groups have been classified as PVTG (formerly known as Primitive Tribal Group/PTG) because they are not only diminishing tribal groups, but they are also constantly facing threats and challenges related to their lifestyle, habitat and health status. They have eventually met the criteria to be classified as PVTG, which include pre-agricultural level of technology, low literacy, economic backwardness and declining population. PVTG members live in 18 states and territories and their health status is alarmingly low, despite the efforts of the Ministry of Tribal Affairs and the National Advisory Council, as well as various schemes and recommendations for development in the areas of education, health, livelihood, skill development, agricultural development, housing and habitat, and cultural preservation. The neglect of maternal and child health, culturally imposed restrictions on family planning services, traditional practices for treating communicable diseases and mental illness, lack of data on social determinants and health aspects, and underutilization of healthcare services indicate that there is a pressing need to address the existing shortcomings among PVTGs in India [6].

### Governmental Initiatives for the Welfare of Tribal Groups

The Ministry of Social Justice and Empowerment was renamed as The Ministry of Tribal Affairs (MTA) in 1999 aiming at focused approach towards the integrated socio-economic development of the ST in a coordinated and planned manner. MTA is the nodal ministry for overall policy planning and coordination of tribal welfare programs. The Ministry is in charge of providing social security and insurance to the poor, as well as organizing and implementing tribal welfare initiatives, which includes research. However, the primary responsibility to promote the interests of ST rests with all Central Ministries. The MTA manages various economic, educational and social development schemes through State Governments and Union Territory (UT) administrations. The Ministry aims to support the welfare of tribes by awarding scholarships for students of different categories, establishing residential schools, supporting Tribal Research Institutes (TRI), providing livelihood support for the tribes and promoting NGO activities for tribal development [31]. Although such efforts are stemmed by the GoI, the objectives and targets often ignore various socio-cultural, political and other administrative barriers existing within the system. So, despite the continuous efforts by the government, these targeted groups spread across the country are still underprivileged and downtrodden from the mainstream of society, evidenced by tremendous disparity in health indicators among tribes and general population for decades, which has not been documented systematically in the national registries [9, 32]. Apart from the Ministry’s activities, India has been implementing the Integrated Tribal Development Projects (ITDP) under the Tribal Sub-Plan (TSP) since the Fifth Five Year Plan, with the specific goals of reducing poverty, improving educational status, controlling tribal family exploitation and preventing atrocities against tribals. In India, there are 193 ITDPs (Integrated Tribal Development Agencies) scattered over 28 states and eight UTs [33].

## Challenges

Due to the disparate geographical locations and population dispersal among India’s tribal communities, the government and other non-governmental organizations (NGOs) working together to uplift them confront numerous hurdles. Many publications and literature compendiums have underlined the following obstacles that stakeholders must overcome in order to improve tribal health in India [6, 34].

### Lack of Health Facilities and Awareness of Health Issues

Lack of awareness or ignorance among tribals regarding many health issues and illnesses often tends to put their lives at risk by delaying the treatment or receiving wrong treatment by untrained traditional healers, and by depending on magical remedial measures. Due to language and cultural hurdles, which are exacerbated by illiteracy and local dialectal barriers, most health communication delivered by healthcare staff is misread or misunderstood by the tribal community. The correspondence between transmitted and received signals is critical in order to avoid negative and counterproductive effects [35]. As the authors have discussed earlier in this article, the communication barrier can be overcome by a cross-cultural relationship between the healthcare professionals and the tribal community [3, 28].

### Lack of Emergency Transportation

There are no well-established roads or modes of transit in most of the tribal hamlets mainly due to their distant location and forest-dwelling customs (out of 145,000 tribal villages in India). In many cases of emergencies, ambulance and doctors might not be able to reach the tribal colonies to transport the patients, due to poor or non-existence of roads [7]. Despite the fact that the schemes such as *Janani Suraksha Yojana* (translated to English as Maternity Safety Scheme) are intended to reduce maternal and neonatal mortality rates, only 54.7% of tribal women have benefited from it as hurdled by poor socio-economic class, backward caste and unskilled type of occupation, which accounts for 27% of home deliveries which adds to the burden of children with low birth weight and other neonatal ailments [6, 17].

### Discriminatory Behaviour by Healthcare Providers

The literature supports the fact that lack of trust and confidence of tribal patients in the public health system is influenced by the discriminative behaviour of healthcare providers. The primitive way of living, eating habits, language and cultural practices among various tribes play a significant role in exhibition of discrimination by healthcare providers towards these vulnerable people. This could affect their health-seeking behaviour as the healthcare staff would be insensitive and unfriendly towards them at healthcare facilities, they may not get proper attention from these staff and the staff could show unwelcoming reaction to indigenous language and their specific appearance, and illiteracy. The cultural gaps between the tribal and general population lead to the exploitation of ST for informal payments for healthcare services and referral to private dispensaries. The discrimination and non-acceptance of their tradition and belief have also made the tribal people to deter the healthcare access and rely on their traditional healers or home remedies [28, 36].

### Financial Constraints

National Scheduled Tribes Finance and Development Corporation (NSTFDC) is an apex organization set up exclusively for the economic development of STs by providing financial assistance at concessional rates of interest. During the year 2019–2020, the corporation has sanctioned financial assistance of 1850 million rupees, covering 54,217 beneficiaries. The corporation had also released 1255 million rupees for the implementation of various schemes till the end of the year 2019 [20]. The outcome of the government schemes and programs for tribal development depends on the socio-economic organization (structure and living conditions of tribal groups), and how effectively the money spent on their welfare has been utilized by them [37].

## Innovations/Opportunities

From the outcomes of various state projects supported by the World Bank, the following innovative strategies can be adopted for the future programs to improve the tribal people’s access to healthcare, to improve the quality of services provided and to mitigate the consequences of the marginalization, imposed by the unfavourable attitude by the mainstream population towards the tribal people [38].

### Creating Awareness About Health Issues

Raising awareness about health and illness, as well as prevention of illness and the promotion of health, is the first step in improving the health outcomes of tribal people. Information, education and communication (IEC) campaigns incorporating various mass and social media and emphasizing the importance of handwashing and hygienic practices, antenatal check-ups, institutional deliveries, immunization, early diagnosis and treatment, and follow-up visits have proven to be effective health promotion interventions in preventing and managing lifestyle diseases and communicable diseases. The programs can incorporate the services of Accredited Social Health Activists (ASHA), through National Health Mission (NHM) to disseminate knowledge through live performances like folk music, puppet shows and magic shows to percolate into their lives. Apart from this strategy, posters, radio talks, brochures, graffiti with a sustainable action plan involving schools, colleges and workplaces in the community would be certainly beneficial to inculcate health literacy among tribal people [38].

### Bringing Health Services to Remote Populations

Through intersectoral coordination and engagement with NGOs, medical outreach camps and mobile clinics may make people’s participation extremely potent through door-to-door canvassing. Preventive and health promotion clinics, such as antinatal clinics and immunization clinics, can be facilitated by the involvement and active participation of healthcare providers in the camps. Basic lab testing, free drugs for the duration of therapy and referral of more difficult patients to higher centres can be facilitated by the concept of satellite clinics in rural areas [38, 39].

### From the People, for the People

The barriers created by the cultural imbalances, language, acceptance and understanding between tribes and healthcare services often reported globally, can be managed up to some extent by promoting the employment opportunity for people from the tribal areas as ASHAs or multipurpose health workers (MPHW) [40]. Counsellors, typically from tribal groups might be stationed at district hospitals in collaboration with local NGOs to advise patients, clarify doctors’ prescriptions, assist patients in taking advantage of welfare programmes and schemes, and counsel them on preventative and promotive health practices.

### Transforming the Behaviour of Healthcare Providers

Acceptability of the tribal population towards the healthcare services chiefly depends on the attitude and approach of the healthcare providers. The non-discriminatory behaviour and understanding the culture and other traditions of the tribal population would chiefly facilitate better acceptance of the healthcare services by them. Behaviour change communication campaigns and organizing meetings and discussions with community leaders would be beneficial for the healthcare providers to understand the tribal community in a better way [28].

### Boosting Economy

Lack of reliable public healthcare services in underserved tribal areas prompted certain tribal welfare projects to partner with NGOs for the provision of free inpatient care by private healthcare establishments to tribal populations. This strategy resulted in the number of inpatients at the hospital facility run by the Nilgiris Waynad Tribal Welfare Society (Tamil Nādu and Kerala states) to increase from seven per month to 47 per month over the two-and-a-half-year period between 2008 and 2010 [7]. Such grants and implementations which were specifically monitored were crucial for the successful execution of welfare programs for tribal populations.

### Ensuring Sustainability

The sustainability of any program depends on meticulous planning, execution and program evaluation. Various programs had been implemented through different 5-year plans, various schemes established for the development of tribal population and financial aid for the upliftment of the poorest categories fail in sustainability as it lacks clarity in objectivity with political and socio-economic fluctuations of the country. The overlooked challenges and opportunities are depicted in Table 3.

**Table 3:** Challenges and opportunities matrix in the implementation of healthcare services among tribes in India

Challenges	Opportunities
Lack of awareness on health issues; poor health-seeking behaviour	Creation of awareness regarding health issues among the community stakeholders who would in turn improve health literacy in their communities and their health-seeking behaviour
Lack of healthcare facilities; inadequate manpower	Bringing health services to remote areas resided by tribal population, motivating the staff delivering healthcare services in these areas
Lack of emergency transportation	Community participation
Discriminatory behaviour by healthcare providers	Transforming behaviour of healthcare providers
Financial constraints	Boosting economy Encouraging sustainability of programs

## Discussion

Tribal populations in India contribute a significant proportion of the total population in contrast with the global scenario. Majority of the problems of tribal populations are being solved by coordinated efforts of governmental and other organizations such as private healthcare agencies and NGOs, which are influenced by a blend of problems. Those problems are deep rooted among the tradition and cultural beliefs of tribal populations itself or associated with the healthcare services delivery in each remote area where those tribals dwell. Many times the burden of illness among tribals is addressed well, yet the barriers and unfavourable attitude of them act as a hindering factor to achieve the goal as it is set for, especially in the delivery of healthcare services among tribals in India.

With the emerging tropical illnesses, endemics and pandemic in the past years, the healthcare burden and challenges in the effective delivery of healthcare services have been escalated. Despite the heterogeneity in the geographical terrain, ethos and tradition, the government has achieved the goal of uplifting the health status of tribal population up to a certain measurable extent [19]. A well-coordinated inter sectorial approach, emphasizing on the sustainable development goals, would certainly help in tackling the existing challenges and improve the health status of tribes in India, as evidenced by a study from Kerala [21]. Recent inputs from Canada support the findings from Kerala, that indigenous led–healthcare practices are effectively beneficial. However, an appropriate government support and intersectoral collaborations would probably aid in execution of better preventive and promotive healthcare services for tribals in India [41]. This is broadly supported by the results of a qualitative study conducted in Nigeria where the participants expressed that the shattered healthcare system and other uncompromised technical aspects repel people from the healthcare services and they mistrust the healthcare system unless and until it progress into a hopeful direction, and this situation drastically reduces the healthcare utilization by the people [42].

Due to a lack of access to healthcare facilities, their rural habitats and socio-cultural discrimination, India’s tribal communities are severely neglected and denied by many healthcare services and they mainly rely on traditional healing practices. This is similar to the findings reported from Congo, with more than 79% of the tribal population following traditional healing practices, neglecting the services offered by healthcare agencies [43]. The Indian government has taken a number of steps to improve the living standards and quality of life of tribal peoples through various schemes and platforms. Tribes have their own culture and tradition, which differs even among subgroups within a tribal group and influences their healthcare beliefs, practices and eventually their utilization of healthcare services. A systematic review on studies conducted among ethnic minorities in China also reveals the factors like inadequate manpower, substandard infrastructure, scattered population and complex mitigation patterns reduced the quality of healthcare services provided to ethnic minority groups, despite the introduction of various policies and schemes by the government [44].

Many deep-rooted issues in the execution of programs are unaddressed as the dearth of data creates the limitation. Healthcare infrastructure and policies related to tribes in India are unable to infiltrate through some of the long-standing barriers and penetrate the healthcare accessibility into the tribal communities. This finding is well supported by a study done among hilly tribals in Thailand, where the distance to healthcare facility, poor socio-economic conditions, poor communication systems, lack of healthcare professionals and policy hurdles played a crucial role in poor access to healthcare services [45]. However, prudent financial, manpower and infrastructure planning could be less superficial, so that the objectives of tribal upliftment programs could be achievable with SMART (Specific, Measurable, Achievable, Realistic and Time bound) programs in the forthcoming 5-year plans.

## Directions for Future Action Plan

The SDGs (1 to10) are actually also focused towards the upliftment of tribal population in all countries, with the goals starting from SDG 1*-*“No Poverty” to SDG 10-“Reduced Inequalities”. However, we assume that a comprehensive understanding and analysis is needed to study the extent of deficiencies existing with respect to the services provided and services received. The barriers in accessing healthcare services should be minimized and the facilitators to be strengthened further. Our narrative review urges the healthcare providers to take up the opportunities to its maximum potential to provide the best community oriented, culturally congruent and need based healthcare services to the tribal people. This further reassures researchers and tribal program coordinators to conduct and facilitate more research among various healthcare issues among the tribal people, especially among PVTGs, that could pave way into the roads that are less travelled.

## Limitation

Though we have made an extensive effort to reiterate the importance of a focussed and program-oriented action plan and interventional strategies that improved the health status of tribal people in India, we were unable to include the scenario of other countries due to the large heterogeneity in the geographical and socio-cultural factors of Indian tribes as compared to those from other selected countries.

## Conclusion

Although India has multifaceted development in many sectors during this millennium, due to lack of access to healthcare facilities, their rural habitats and socio-cultural discrimination, India’s tribal communities are severely neglected and deprived of many healthcare services. The government has initiated a lot of programs to improve their living standards and quality of life through various schemes and platforms. Many deep-rooted issues in the execution of programs are unaddressed due to paucity of data. Healthcare infrastructure and policies related to tribes in India are unable to infiltrate through their culture as well as language barriers and often fail to penetrate into the tribal communities. For example, the healthcare workers including ASHA, being in the grass root level of health-promoting activities, do not completely understand the colloquial language spoken by tribes staying in forest or hilly areas. Hence, having financial resources with trained manpower oriented towards indigenous culture, appropriate infrastructure planning and tribal upliftment programs are crucial in closing the existing gaps and enhance the health status of tribes in India.

## Data Availability

Not applicable.

## References

[1] Europe and Asia aging rapidly, while Africa is home to the world’s largest youth population. World Population Datasheet. 2019. http://www.prb.org/worldpopdata/ Accessed 19 June 2022.

[2] Scheduled casts and scheduled tribes. Office of the Registrar General and Census Commissioner, India.2001. https://www.censusindia.gov.in/Census_And_You/scheduled_castes_and_sceduled_tribes.aspx. Accessed 19 June 2022.

[3] Statistical profile of scheduled tribes in India. Ministry of Tribal Affairs. Government of India. 2013. https://tribal.nic.in/ST/StatisticalProfileofSTs2013.pdf. Accessed 19 June

[4] Special representation in services for SC/ ST. Department of Personnel and Training, Government of India. 2019. https://dopt.gov.in/sites/default/files/ch-11.pdf. Accessed 19 June 2022.

[5] Scheduled tribes in India. Census of India 2011. Ministry of Tribal Affairs, Government of India. 2011. https://tribal.nic.in/Statistics.aspx. Accessed 19 Jan 2022.

[6] Tribal health in India, bridging the gap and a roadmap for the future- executive summary and recommendations. Ministry of Health and Family Welfare and Ministry of Tribal Affairs, Government of India. 2018. https://www.nhm.gov.in/nhm_components/tribal_report/Executive_Summary.pdf. Accessed 19 June 2022.

[7] Mavalankar D. Doctors for tribal areas: issues and solutions. Indian J Community Med. 2016;41:172–6. 10.4103/0970-0218.183587. 27385868 10.4103/0970-0218.183587 PMC4919928

[8] Baethge C, Goldbeck-Wood S, Mertens S. SANRA—a scale for the quality assessment of narrative review articles. Res Integr Peer Rev. 2019;4(5). 10.1186/s41073-019-0064-8. 10.1186/s41073-019-0064-8 PMC6434870 30962953

[9] Mohindra K, Labonté R. A systematic review of population health interventions and Scheduled Tribes in India. BMC Public Health. 2010;10:438. 10.1186/1471-2458-10-438. 20659344 10.1186/1471-2458-10-438 PMC2919477

[10] National Family Health Survey (NFHS-4). (2016). India fact sheet. Ministry of Health and Family Welfare, Government of India. http://rchiips.org/NFHS/pdf/NFHS4/India.pdf. Accessed 2 Feb 2023.

[11] Sahu D, Nair S, Singh L, Gulati BK, Pandey A. Levels, trends & predictors of infant & child mortality among Scheduled Tribes in rural India. Indian J Med Res. 2015;141:709–19. 26139791 10.4103/0971-5916.159593 PMC4510772

[12] Thomas BE, Adinarayanan S, Manogaran C, Swaminathan S. Pulmonary tuberculosis among tribals in India: a systematic review & meta-analysis. Indian J Med Res. 2015;141:614–23. 26139779 10.4103/0971-5916.159545 PMC4510760

[13] Rao VG, Bhat J, Yadav R, Muniyandi M, Sharma R, Bhondeley MK. Pulmonary tuberculosis - a health problem amongst Saharia tribe in Madhya Pradesh. Indian J Med Res. 2015;141:630–5. 26139781 10.4103/0971-5916.159560 PMC4510762

[14] Purty AJ, Mishra AK, Chauhan RC, Prahankumar R, Stalin P, Bazroy J. Burden of pulmonary tuberculosis among tribal population: a cross-sectional study in tribal areas of Maharashtra, India. Indian J Community Med. 2019;44:17–20. 30983707 10.4103/ijcm.IJCM_120_18 PMC6437794

[15] Devarapalli SVSK, Kallakuri S, Salam A, Maulik PK. Mental health research on scheduled tribes in India. Indian J Psychiatry. 2020;62:617–30. 33896966 10.4103/psychiatry.IndianJPsychiatry_136_19 PMC8052874

[16] McCalman J, Bainbridge R, Percival N, Tsey K. The effectiveness of implementation in Indigenous Australian healthcare: an overview of literature reviews. Int J Equity Health. 2016;15:47. 10.1186/s12939-016-0337-5. 26965040 10.1186/s12939-016-0337-5 PMC4787175

[17] Kumar MM, Pathak VK, Ruikar M. Tribal population in India: a public health challenge and road to future. J Family Med Prim Care. 2020;28:508–12. 10.4103/jfmpc.jfmpc_992_19. 10.4103/jfmpc.jfmpc_992_19 PMC7113978 32318373

[18] Rural Health Statistics 2021-22. Ministry of Health and Family Welfare. Government of India. https://hmis.mohfw.gov.in/downloadfile?filepath=publications/Rural-Health-Statistics/RHS%202021-22.pdf. Accessed 15 January 2023.

[19] Ministry of tribal affairs. Annual Report. New Delhi. Government of India. 2020. Available from https://tribal.nic.in/writereaddata/AnnualReport/AREnglish1920. Accessed 17 Feb 2023.

[20] Kushwah VS, Sisodia R, Bhatnagar C. Magico-religious and social belief of tribals of district Udaipur, Rajasthan. J Ethnobiol Ethnomed. 2017;13:69. 10.1186/s13002-017-0195-2. 29191222 10.1186/s13002-017-0195-2 PMC5709986

[21] George MS, Davey R, Mohanty I, et al. “Everything is provided free, but they are still hesitant to access healthcare services”: why does the indigenous community in Attapadi, Kerala continue to experience poor access to healthcare? Int J Equity Health. 2000. 10.1186/s12939-020-01216-1. 10.1186/s12939-020-01216-1 PMC7320563 32590981

[22] Burke NJ, Bird JA, Clark MA, Rakowski W, Guerra C, Barker JC, Pasick RJ. Social and cultural meanings of self-efficacy. Health Educ Behav. 2009;36:111S–28S. 10.1177/1090198109338916. 19805794 10.1177/1090198109338916 PMC2921833

[23] Maher P. A review of ‘Traditional’ aboriginal health beliefs. Aust J Rural Health. 1997;229–36. 10.1046/j.1440-1584.1999.00264.x. 10.1046/j.1440-1584.1999.00264.x 10732513

[24] Rahman SA, Kielmann T, McPake B, Normand C. Healthcare-seeking behaviour among the tribal people of Bangladesh: can the current health system really meet their needs? J Health Popul Nutr. 2012;30:353–65. 10.3329/jhpn.v30i3.12299. 23082637 10.3329/jhpn.v30i3.12299 PMC3489951

[25] Vijayakumar KN, Gunasekaran K, Sahu SS, Jalbulingam P. Knowledge, attitude and practice on malaria: a study in tribal belt of Orissa State, India with reference to use of long-lasting treated mosquito nets. Acta Trop. 2009;112:137–42. 19631184 10.1016/j.actatropica.2009.07.011

[26] Dhiman RC, Shahi B, Sharma N, Nanda N, Khargiwarkar VN, et al. Persistence of malaria transmission in a tribal area in Maharashtra, India. Current Sci. 2005;88:475–8.

[27] Priyadarshini P, Abhilash PC. Promoting tribal communities and indigenous knowledge as potential solutions for the sustainable development of India. Environ Dev. 2019;32:100459. 10.1016/j.envdev.2019.100459.

[28] Wilson AM, Kelly J, Jones M, O’Donnell K, Wilson S, Tonkin E, Magarey A. Working together in Aboriginal health: a framework to guide health professional practice. BMC Health Serv Res. 2020;20(1):601. 10.1186/s12913-020-05462-5. 32611413 10.1186/s12913-020-05462-5 PMC7329497

[29] Thomas ST, Thomas ET, McLean M, et al. Paving the way to achieving the United Nations Sustainable Development Goals for women from Indigenous communities: lessons from Attappady, India. Discov Sustain. 2021:2. 10.1007/s43621-021-00009-y.

[30] Lemaster PL, Connel CM. Health education interventions among Native Americans: a review and analysis. Health Educ Quart. 1994;21:521–38. 10.1177/109019819402100413 7843981

[31] Jordan JB. Mental health considerations with the Yupik Eskimos. Alaska Med. 1997;39:67–71. 9368422

[32] Ministry of Tribal Affairs, Government of India. Historical background. Available from https://tribal.nic.in/aboutTheMinistry.aspx. Accessed 28 Oct 2022.

[33] Sarkar AK, Singha S. Factors influencing health of the Santals: a study of selected villages of Birbhum. Int J Community Soc Dev. 2019;1:58–74. 10.1177/2516602619826261.

[34] Ministry of Tribal Affairs. Government of India. Constitutional and legal matters, List of ITDPs/ITDAs/MADA Pockets, Clusters. https://tribal.nic.in/clm.aspx. Accessed 28 Oct 2022.

[35] Purushottam DV. Understanding the Indian Tribal life and their issues. Int J Adv Res. 2017;5:1588–95.

[36] Cho H, Salmon CT. Unintended effects of health communication campaigns. J Comm. 2007;57:293–317. Accessed 24 Apr 2023

[37] Thummapol O, Barton S, Park T. Healthcare access experiences among indigenous women in Northern Rural Thailand: a focused ethnographic study. Cent Asian J Glob Health. 2018. 10.5195/cajgh.2018.328. 10.5195/cajgh.2018.328 PMC6393055 30863666

[38] Sonowal CJ. Factors affecting the nutritional health of tribal children in Maharashtra. Stud Ethno-Med. 2010;4:21–36. 10.1080/09735070.2010.11886359.

[39] Mallya SD, Shreedhar S, Sudhakaran D, Aravindhkumar B, Nair S, Shetty RS. Health status of Koraga community: a pilot study among a particularly vulnerable tribal group of Udupi District, Karnataka, India. Indian J Med Res. 2022;156:275–83. 10.4103/ijmr.ijmr_3209_21. 36629187 10.4103/ijmr.ijmr_3209_21 PMC10057362

[40] Assessing the research on home visiting program models implemented in tribal communities—Part 1: Evidence of effectiveness. https://www.acf.hhs.gov/opre/resource/assessing-research-home-visiting-program-models-implemented-tribal-communities. Accessed June 08, 2022.

[41] Allen L, Hatala A, Ijaz S, Courchene ED, Bushie EB. Indigenous-led health care partnerships in Canada. CMAJ. 2020;192:E208–16. 10.1503/cmaj.190728. 32122977 10.1503/cmaj.190728 PMC7055951

[42] Ogueji IA, Ogunsola OO, Abdalla NM, et al. Mistrust of the Nigerian health system and its practical implications: qualitative insights from professionals and non-professionals in the Nigerian health system. J Public Health (Berl.). 2023. 10.1007/s10389-022-01814-z.

[43] Mutombo CS, Bakari SA, Ntabaza VN, Nachtergael A, Lumbu J-BS, Duez P, et al. Perceptions and use of traditional African medicine in Lubumbashi, Haut-Katanga province (DR Congo): a cross-sectional study. PLoS One. 2022;17(10):e0276325. 10.1371/journal.pone.0276325. 36256659 10.1371/journal.pone.0276325 PMC9578634

[44] Huang Y, Shallcross D, Pi L, Tian F, Pan J, Ronsmans C. Ethnicity and maternal and child health outcomes and service coverage in western China: a systematic review and meta-analysis. Lancet Glob Health. 2018;6(1):e39–56. 10.1016/S2214-109X(17)30445-X. 29153766 10.1016/S2214-109X(17)30445-X

[45] Moonpanane K, Pitchalard K, Thepsaw J, Singkhorn O, Potjanamart C. Healthcare service utilization of hill tribe children in underserved communities in thailand: barriers to access. BMC Health Serv Res. 2022;22(1):1114. 10.1186/s12913-022-08494-1. 36050759 10.1186/s12913-022-08494-1 PMC9438234

